# Exhaled Nitric Oxide as a Biomarker in COPD and Related Comorbidities

**DOI:** 10.1155/2014/271918

**Published:** 2014-02-27

**Authors:** Mario Malerba, Alessandro Radaeli, Alessia Olivini, Giovanni Damiani, Beatrice Ragnoli, Paolo Montuschi, Fabio L. M. Ricciardolo

**Affiliations:** ^1^Department of Internal Medicine, University of Brescia and Civil Hospital of Brescia, Piazza Spedali Civili 1, 25100 Brescia, Italy; ^2^Department of Dermatology, Civil Hospital of Brescia, Piazza Spedali Civili 1, 25100 Brescia, Italy; ^3^Department of Pharmacology, Faculty of Medicine, Catholic University of the Sacred Heart, Largo Francesco Vito 1, 00198 Rome, Italy; ^4^Department of Clinical and Biological Sciences, University of Torino, San Luigi Hospital, Regione Gonzole 10, 10043 Orbassano, Italy

## Abstract

Chronic Obstructive Pulmonary Disease (COPD) is defined as a disease characterized by persistent, progressive airflow limitation. Recent studies have underlined that COPD is correlated to many systemic manifestations, probably due to an underlying pattern of systemic inflammation. In COPD fractional exhaled Nitric Oxide (FeNO) levels are related to smoking habits and disease severity, showing a positive relationship with respiratory functional parameters. Moreover FeNO is increased in patients with COPD exacerbation, compared with stable ones. In alpha-1 antitrypsin deficiency, a possible cause of COPD, FeNO levels may be monitored to early detect a disease progression. FeNO measurements may be useful in clinical setting to identify the level of airway inflammation, *per se* and in relation to comorbidities, such as pulmonary arterial hypertension and cardiovascular diseases, either in basal conditions or during treatment. Finally, some systemic inflammatory diseases, such as psoriasis, have been associated with higher FeNO levels and potentially with an increased risk of developing COPD. In these systemic inflammatory diseases, FeNO monitoring may be a useful biomarker for early diagnosis of COPD development.

## 1. Introduction 

As is well known, chronic obstructive pulmonary disease (COPD) is simply considered a lung disease characterised by the presence of fixed and progressive airflow limitation derived from airway inflammation/remodelling associated with parenchymal destruction so-called pulmonary emphysema. However, in most of COPD patients the disease coexists with several other systemic manifestations which can make health-related quality of life worse and increase mortality [1]. Thus, COPD could no longer be defined as a disease restricted to the lung but might be considered part of a complex chronic systemic disease previously defined as “chronic systemic inflammatory syndrome” [2].

The best-recognised comorbidities in COPD include lung cancer, cardiovascular diseases, malnutrition involving primarily the loss and dysfunction of skeletal muscles, osteoporosis, anaemia, diabetes, increased gastroesophageal reflux, metabolic syndrome, obstructive sleep apnoea, depression, and anxiety. Comorbidities can be classified in conditions that share pathogenetic mechanisms with COPD (e.g., smoking-related diseases such as ischemic heart disease and lung cancer), conditions that complicate COPD (such as osteoporosis and sarcopenia), and conditions that are simply associated with COPD for epidemiologic reasons (like glaucoma and obstructive sleep apnoea) [3].

In COPD patients, the high frequency of concurrent diseases may be largely explained by the old age of the majority of patients and by cigarette smoke exposure, the major risk factor for COPD, many other chronic diseases, and certain cancers. Smoking triggers a local inflammatory response throughout the whole tracheobronchial tree, and pathologic changes, a characteristic of COPD, are found in the proximal large airways, peripheral small airways, lung parenchyma, and pulmonary vasculature [4]. Apart from these local effects, smoking may significantly contribute to or cause systemic inflammation including the stimulation of the hematopoietic system with polymorph nuclear leukocytes release, the generation of systemic oxidative stress, and the endothelial dysfunction of peripheral vessels [4]. These systemic effects due to smoking may account for the frequent concurrent presence of other chronic illnesses such as cardiovascular diseases and metabolic disorders in COPD patients [4].

Furthermore, one-half of all people aging more than 65 years have at least three chronic medical conditions, and aging itself is associated with a chronic low-grade inflammatory status [5]. Thus, the theory that systemic inflammation is the common driver of chronic diseases would explain the high prevalence of chronic diseases with increasing age, so-called “inflammaging” [5]. Concerning the observed associations between COPD and its comorbidities, there are two possible explanations: the systemic “spill-over” of the inflammatory and reparatory events occurring in COPD lungs with a central role of the disease in the process and the “systemic” inflammatory state due to multiple organ compromise which includes also COPD pulmonary manifestations [2, 6]. Patients with COPD show systemic inflammation, especially related to disease severity and exacerbations, that can be measured as increment of circulating cytokines (IL-6, TNF-*α*), chemokines (IL-8), and acute phase proteins (C-reactive protein (CRP) and surfactant protein D) or abnormalities in circulating cells [7]. One of the most important pathogenetic mechanisms of COPD is oxidative stress associated with inflammatory cellular infiltration (macrophages, neutrophils, and lymphocytes CD8) that in conjunction with an altered release of endogenous nitric oxide (NO) may provoke the formation of “nitrative stress” [6].

## 2. Nitric Oxide and COPD 

Endogenous nitric oxide (NO) is a gaseous signaling molecule produced by residential and inflammatory cells in both large and peripheral airways/alveoli. NO plays an important role in regulating airway and vascular function and is generated by three isoforms of NO synthases (neuronal NOS (nNOS, NOS1), endothelial NOS (eNOS, NOS3), and inducible NOS (iNOS, NOS2)) with different expression and pathophysiologic roles in the airways. In particular, iNOS is not constitutively expressed but is induced by several stimuli including endogenous mediators (chemokines and cytokines) and exogenous factors (bacterial toxins, viral infection, allergens, environmental pollutants, etc.) [8]. NO is generated by the conversion of L-arginine to L-citrulline and during inflammation, due to iNOS induction, large amounts of NO are produced which may exert proinflammatory effects [9].

Corticosteroids directly suppress iNOS in rodent cells but do not directly inhibit iNOS expression in human airway epithelial cells [10]. The increase in NO in exhaled breath in asthma is presumed to originate from increased iNOS expression in the respiratory tract, although cNOS isoforms (nNOS and eNOS) may also contribute. Increased iNOS expression is found in airway epithelial cells of patients with asthma and is reduced by inhaled corticosteroids [11]. Increased iNOS expression is found in central and small airways and in peripheral lung of COPD patients [12, 13]; however, there was no effect of high-dose ICS on exhaled nitric oxide at both airway and alveolar compartments [14] suggesting that probably in COPD iNOS is not the main source of NO. In other studies, selective inhibitors of iNOS reduce FeNO in asthmatic patients and also in normal subjects [15–17] but have less effect on COPD patients. In a recent study, Brindicci et al. showed that nNOS expression and activity are increased in COPD according to disease severity suggesting that in these patients the increased peripheral NO may be derived from nNOS [12].

Oxidative stress generates superoxide anions and in combination with NO may result in the formation of the highly reactive species peroxynitrite, which is increased in exhaled breath condensate and airway mucosa of COPD patients [13–18] and removes NO from the gaseous phase so that its concentration in the airways is reduced when there is a high level of oxidative stress, as in COPD patients [10].

## 3. FeNO and COPD 

FeNO measurements have been considered a surrogate for eosinophilic airway inflammation, especially in asthma. In most mild asthmatics, high FeNO at 50 mL s^−1^ (>45 ppb) has been regarded as a marker for steroid responsiveness [19, 20] including improvement in spirometry and airway hyperresponsiveness [21]. The ATS clinical practice guideline for exhaled nitric oxide in asthma concluded that add-on FeNO monitoring provides potential easier detection of eosinophilic airway inflammation and likelihood of corticosteroid responsiveness [22].

FeNO levels in COPD are conflictual [8], but it seems that smoking habits and disease severity are the most important factors influencing exhaled NO levels in these patients [23]. Current smokers [24] and severe COPD (particularly in combination with *cor pulmonale*) [25] show lower levels of exhaled NO than ex-smokers and mild/moderate COPD (Table 1). Increased exhaled NO levels have been reported in hospitalized patients during an exacerbation of COPD (Table 1) [26]. Interestingly, exhaled NO levels returned to control values only months after discharge of those steroid-treated patients, suggesting different inflammatory mechanisms in COPD compared with the highly steroid-sensitive asthmatics [26].

In COPD patients, the magnitude of the NO signal is considerably less than in asthma and, most importantly, the major causative agent, cigarette smoke, dramatically masks any tendency towards a disease related rise in exhaled NO levels. This may have consequences not only for monitoring patients with COPD, but also for the natural history and prognosis of the disease. The positive relationship between exhaled NO levels and FEV_1_ is in keeping with the hypothesis that endogenous NO represents an important protective mechanism. This could be particularly relevant in patients with COPD who may require local NO release for antimicrobial host defence or preservation of ventilation/perfusion matching within the lung [23].

Several studies [27–31] showed that elevated FeNO in COPD may also be a variable signal for increased spirometric response to ICS. Moreover FeNO is increased in patient with COPD exacerbation, compared to the levels observed in patients with stable COPD [32]. In a recent study, Soter at al. demonstrated that FeNO is a good biomarker of eosinophilic inflammation in COPD patients with exacerbation [33].

In conclusion, in COPD the role of FeNO monitoring to therapeutic intervention is still unclear with respect to clinical relevance in particular because of the absence of randomized, double blind, control studies.

### 3.1. FeNO in Alpha-1 Antitrypsin (AAT) Deficiency

Alpha-1 antitrypsin (AAT) deficiency is a genetic disorder due to homozygosity or heterozygosity for the protease inhibitor (Pi) Z allele, and these two genetic phenotypes are, respectively, characterized by a severe reduction (PiZZ) and a lower reduction (PiMZ) of plasma levels of AAT than in normal subjects. The homozygotic form of the disease is considered to be an important risk factor for developing COPD [34].

Severe AAT deficiency (PiZZ) is characterized by lower FeNO level (Table 1) compared to healthy nonsmokers and COPD patients, which is correlated to the pulmonary function impairment which is a characteristic of this kind of patients [35, 36]. While, PiMZ subjects show increased FeNO levels (Table 1) compared to COPD patients and healthy controls [37] and FeNO levels are related to the reduced concentration of AAT in plasma [38]. In these patients FeNO measurement may be very important to monitor a possible progression of airways inflammation to COPD.

### 3.2. FeNO in Pulmonary Arterial Hypertension

Pulmonary arterial hypertension (PAH) is characterized by an elevation of the pulmonary arterial pressure and an increased pulmonary vascular resistance, leading to decline in cardiopulmonary function and premature death [39].

PAH is commonly caused by an underlying pulmonary or systemic disease, whilst idiopathic PAH (IPAH) is referred to PAH when diagnosed in the absence of an identifiable cause or disease.

IPAH is the most studied form of PAH, characterized by endothelial and smooth muscle cell proliferation, medial hypertrophy, and thrombosis in situ [40]. The elevated vascular resistance probably is due to an imbalance between local vasodilators and vasoconstrictors, associated with cellular proliferation and vascular remodeling. NO is one of the important pathophysiologic mediators of pulmonary vascular resistance [41, 42]. NO produced in the upper and lower airways by NOS2 is able to affect the pulmonary vascular tone together with the NO produced by NOS3 in the vascular endothelium [43]. Once produced NO is highly diffusible and activates soluble guanylate/cyclase in pulmonary vascular smooth muscle cells to produce guanosine 39-59-cyclic monophosphate, inducing vascular smooth muscle relaxation and then vasodilation [44]. Patients with PAH show low FeNO values (Table 1) [44, 45]. Patients with PAH also show lower concentrations of NO than normal in the BAL fluid, inversely related to the degree of pulmonary hypertension [46]. Therefore, replacement of NO seems to work well in treating PAH [42]. Phosphodiesterase type 5 inhibitors, preventing the collapse of the NO effector molecule 39,59-cyclic guanosine monophosphate, prolong NO mediated vasodilatation [47, 48]. Prostacyclins and endothelin receptor antagonists also are able to reduce the NO deficiency state in patients with PAH [49]. PAH patients who respond to therapy show higher FeNO levels compared with those who do not change their FeNO levels in response to therapy [47]. The presence of reduced FeNO levels in patients with PAH and the increase after treatment suggest that serial monitoring of FeNO in these patients may be useful.

### 3.3. FeNO in Systemic Sclerosis (SSC) with Pulmonary Arterial Hypertension

Systemic sclerosis (SSc) is a connective tissue disorder of unknown etiology, characterized by a multisystemic involvement, and that is often complicated by pulmonary involvement [50], especially PAH with or without interstitial lung disease (ILD) [51]. The mechanisms underlying pulmonary involvement of SSc are unknown, but endothelial changes, both morphological and functional, may be pathogenetically important [52]. Moreover, in these patients inflammation probably promotes the development of pulmonary fibrosis [53]. NO can be implicated in the pathogenesis of both PAH and ILD. In fact, NO plays an important role in maintaining the low resistance of the pulmonary circulation [54], so if basal production of NO mediated by eNOS is low, it could promote vasoconstriction and vascular wall thickening, bringing to PAH [55]. Moreover iNOS is stimulated by inflammatory cytokines [56]; the presence of an inflammatory pattern, such as in SSc, is linked to an increase of iNOS activation; and NO proinflammatory and cytotoxic effects could promote ILD [57]. A recent study investigated the value of FeNO in a group of 50 SSc patients, with the diagnosis based on American Rheumatism Association criteria [58], compared with 40 healthy subjects [45]. The FeNO levels were higher in SSc patients than in control subjects (Table 1), and lower in patient with ILD and/or PAH than in those without PAH, moreover there was an inverse correlation between the severity of pulmonary artery pressure and FeNO levels.

### 3.4. FeNO in Cardiovascular Diseases

#### 3.4.1. Heart Failure

It has been hypothesized that NO provides an important role in heart failure (HF) in either the pathogenesis or disease progression, although the precise role played by NO is complex [59]. In particular, NO release from the vascular endothelium in patients with heart failure may help to maintain tissue perfusion by reducing the vasoconstriction induced by various neurohumoral factors; moreover NO is able to modulate cardiac contractility [59].

In patients with chronic HF, deregulated systemic NO production due to endogenous inhibitors of NO synthases such as asymmetric dimethylarginine (ADMA) has been related to both systolic and diastolic dysfunction in addition to poor long-term adverse outcomes [60].

Several studies in symptomatic HF patients at rest have reported variable FeNO levels compared with normal control subjects, and the results in literature are conflicting. Higher levels of FeNO were observed in decompensated HF resting (Table 1) compared with compensated and resting FeNO decreased after lowering of left ventricular filling pressures [61]. Higher FeNO levels have been observed after exercise in stable chronic HF patients (Table 1) compared with control subjects [62, 63].

Patients who fail to raise FeNO during exercise have been related to a higher long-term mortality rate [64]. Accordingly a potential role of NO production as a compensatory response to the increased pressure in the pulmonary venous circulation has been hypothesized.

As mentioned above, several FeNO studies have shown conflicting results regarding FeNO levels at rest and exercise. These contradictory reports can probably be explained by the difference in the populations studied and the use of different techniques for FeNO analysis; moreover the type of cardiomyopathy is rarely reported, and it can be hypothesized that ischemic heart disease could be much different in terms of NO production than idiopathic dilatative heart disease. However, more recent reports seem to indicate that FeNO levels increased after exercise in chronic compensated HF patients [63]. This seems to be related to the degree of pulmonary congestion and hypertension; probable increased activity of pulmonary endothelial NO synthases appears in response to improved flow, in the pulmonary vasculature taking place during exercise, as a compensatory mechanism. Consequently, higher FeNO may represent a compensatory mechanism in response to high-flow conditions [65].

#### 3.4.2. FeNO in Atherosclerosis and Cardiovascular Risk Factors

Endothelial NO seems to play a key role in the atherogenic process platelet adhesion and aggregation, expression of adhesion molecule and chemokine production, and inflammatory cell infiltration together with smooth muscle cell migration and proliferation [66, 67].

This central balancing function of NO is hindered during the atherosclerotic process, in which endothelial damage causes the decline in bioactivity of endothelial nitric oxide synthase and consequently impaired release of NO. Furthermore, ischemic heart disease (IHD) is characterized by a low-grade systemic inflammation and associated with increased oxidative stress; these factors enhance the inactivation of NO at endothelial level in the arterial wall and could lead to diminished systemic bioavailability of NO [68]. Therefore, it is possible to hypothesize that, because NO production is reduced and its inactivation increased in dysfunctional atherosclerotic endothelium, atherosclerosis is inversely associated with NO and FeNO (Table 1). Consequently a recent paper [69] observed that two common atherosclerosis risk factors, triglycerides serum levels and hemoglobin/A1c (a marker of hyperglycaemic metabolism), were inversely associated with FeNO levels in patients with IHD. This can be explained by the reduced eNOS and the enhanced NO degradation due to endothelial damage, characteristics of atherosclerosis [69].

### 3.5. FeNO in Psoriasis

Psoriasis vulgaris is a multisystem common dermatologic disease characterized by chronic inflammatory pathogenesis [70]. The prevalence in adults ranged from 0,91% in USA to 8,5% in Norway, while Italy with the 2% was in the middle [71]. In literature, psoriasis is also related to several comorbidities; these patients were more likely to have diabetes, dyslipidemia, hypertension, a history of myocardial infarction, inflammatory arthritis, obesity, and metabolic syndrome than control groups [72]. Two recent studies evaluated the correlation between COPD and psoriasis, concluding that there is a significant correlation between these two diseases. A large, population-based case-control Israeli study by Dreiher et al. [73], including 12.502 psoriasis cases and 24.287 controls, demonstrated that the prevalence of COPD was significantly higher in patients with psoriasis (5,7% versus 3,6%, *P* < 0,001 OR = 1,63). Another study, conducted in Taiwan, on 10.480 patients with psoriasis, underlined that psoriasis patients are at a greater risk of developing COPD, with significantly lower COPD-free survival rates than the comparison cohort [74].

Recent developments described psoriasis pathophysiology as mainly directed by Th1 and Th17 cells which provoke a skin barrier dysfunction [75]. In literature, NO production in skin cells was demonstrated physiologically for several cytotypes such as keratinocytes, fibroblasts, and melanocytes [76]. In psoriasic lesions, an overexpression of iNOS is associated with a compensatory increase of arginase 1 enzyme which may reduce the NO availability [77]. On the other hand, direct measurements of NO production in psoriasic lesions did not find any evidence of a competitive inhibition [78]. However, in psoriasis patient's serum Gabr et al. discovered an increase of NO levels that correlated with the PASI (Psoriasis Area Severity Index) [79]. So this study was performed in order to detect if FeNO measurement is useful in revealing earlier the pulmonary involvement in this disease. The preliminary analysis about FeNO levels in psoriasic patients pointed out the strong correlations between the active disease and an increased level of FeNO (Table 1), suggesting a lung involvement.

## 4. Conclusions 

New evidence concerning FeNO in relation to COPD and the best-recognized comorbidities collectively highlights the following potential roles of this biomarker in monitoring (1) the stability of COPD; (2) the response to therapy in heart failure and in PAH patients; (3) the possible progression to COPD of other diseases like alpha-1 antitrypsin deficiency and psoriasis. These latter aspects may be useful to integrate the clinical evaluation of patients with COPD and comorbidities.

Since FeNO levels are strongly affected by cigarette smoking and many COPD patients are current smokers, the usefulness of FeNO measurement can be limited. In COPD patients, the role of add-on FeNO monitoring to therapeutic intervention is less clear with respect to clinical benefits, especially in the absence of conclusive double-blind, randomized, control studies. Therefore, routine monitoring of FeNO in COPD is less established than in asthma as noted in the recent ATS Clinical Guidelines.

## Figures and Tables

**Table 1:** Trend of FeNO levels in COPD and related comorbidities.

Disease/condition	FeNO levels
Smoking habit	↓
Severe COPD	↓
Stable COPD	=
Exacerbation of COPD	↑
AAT deficiency PiZZ	↓
AAT deficiency PiMZ	↑
PAH	↓
Systemic sclerosis	↑
Decompensated HF	↑
After exercise in stable HF	↑
Atherosclerosis	↓
Psoriasis	↑

↑: >20 ppb; =: 10–20 ppb; ↓: <10 ppb.

## Abbreviations

NO: Nitric oxide
NOS: Nitric oxide synthase
nNOS: Neuronal NOS
iNOS: Inducible NOS
eNOS: Endothelial NOS
FeNO: Exhaled nitric oxide fraction
ATS: American Thoracic Society
CS: Corticosteroids
COPD: Chronic obstructive pulmonary disease
CF: Cystic fibrosis
AAT: Alpha-1 antitrypsin
SSc: Systemic sclerosis
PAH: Pulmonary arterial hypertension
ILD: Interstitial lung disease
HF: Heart failure
IHD: Ischemic heart disease
PS: Psoriasis
PASI: Psoriasis area severity index
FEV_1_: Forced expiratory volume in the 1st second.
